# Shape Similarity Measurement for Known-Object Localization: A New Normalized Assessment

**DOI:** 10.3390/jimaging5100077

**Published:** 2019-09-23

**Authors:** Baptiste Magnier, Behrang Moradi

**Affiliations:** IMT Mines Alès, LGI2P, 6. Avenue de Clavières, 30100 Alès, France; behrang.moradi@mines-ales.fr

**Keywords:** distance measures, contours, shape, pose evaluation

## Abstract

This paper presents a new, normalized measure for assessing a contour-based object pose. Regarding binary images, the algorithm enables supervised assessment of known-object recognition and localization. A performance measure is computed to quantify differences between a reference edge map and a candidate image. Normalization is appropriate for interpreting the result of the pose assessment. Furthermore, the new measure is well motivated by highlighting the limitations of existing metrics to the main shape variations (translation, rotation, and scaling), by showing how the proposed measure is more robust to them. Indeed, this measure can determine to what extent an object shape differs from a desired position. In comparison with 6 other approaches, experiments performed on real images at different sizes/scales demonstrate the suitability of the new method for object-pose or shape-matching estimation.

## 1. Introduction and Motivations

Representing an object shape is extremely useful for specific industrial and medical inspection tasks. When a shape is aligned, under supervision, with a reference model, a wide variety of manipulations can arise/be used. Contrary to region-based methods [1], edge-based representation remains a set of methods only exploiting information about shape boundaries. The assessment of acquired features (contours) in a candidate image compared to an ideal contour map model is therefore one approach to the supervised assessment of shape depiction. This paper presents a new approach for the measurement of a contour-based object pose, which is normalized. It follows on from a talk given by the research team in [2], dealing with the subject more thoroughly and in greater detail. The proposed measurement evaluates an estimated supervised score for the shape representation based on the weights created by both false positive and false negative edge pixels. In this context, normalization is highly appropriate for interpreting an algorithm result. Normalization is a technical operator that can determine when a score is suitable in function of the intended operation: if the score is near 1, the action is deemed to be good, whereas a score close to 0 indicates an inappropriate initiative. There exist several techniques to assess a binary shape; usually, they are used in the edge detection evaluation framework. However, the existing normalized methods suffer from various drawbacks: either they consider spurious points (false positives) or they record only missing ones (false negative) and their associated distances. The new method applies various strategies to normalize and reliably assess the contour-based localization of objects. First, misplaced pixels are penalized as a function of their distances from where they should be localized. Secondly, the normalization term is pondered using the number of false positive and false negative points.

The next section is devoted to existing shape-based normalized measures. This demonstrates the advantage of considering distance pixels instead of counting only false positives and false negatives. Moreover, in this current section, the drawbacks of the different measures are shown and detailed, further supporting the choice of the new normalized measure.

The last part of this paper is dedicated to experimental evaluations and results. Experiments are performed on synthetic and real images, where the desired shapes suffer from rotation, translation, or scale changes. The normalization is valuable and robust, it obtains a similar movement evaluation even when a scale change appears. Eventually, as opposed to the 6 other compared normalized measures, the new method calculates a coherence score to qualify the possibility of correct object pose.

## 2. On Existing Normalized Measures

**Figure 1:**
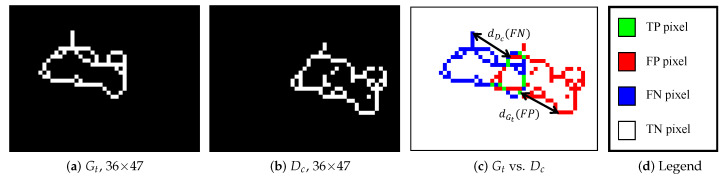
Example a ground truth $G_{t}$ and a desired contour $D_{c}$. For each FN point, the minimum distance between the considered FN and $D_{c}$ is recorded, called $d_{D_{c}}\left(FN\right)$. For each FP point, the minimum distance between the considered FP and $G_{t}$ is recorded, called $d_{G_{t}}\left(FP\right)$. Please note that for a TP pixel, both $d_{D_{c}}\left(TP\right)=0$ and $d_{G_{t}}\left(TP\right)=0$.

**Figure 2:**
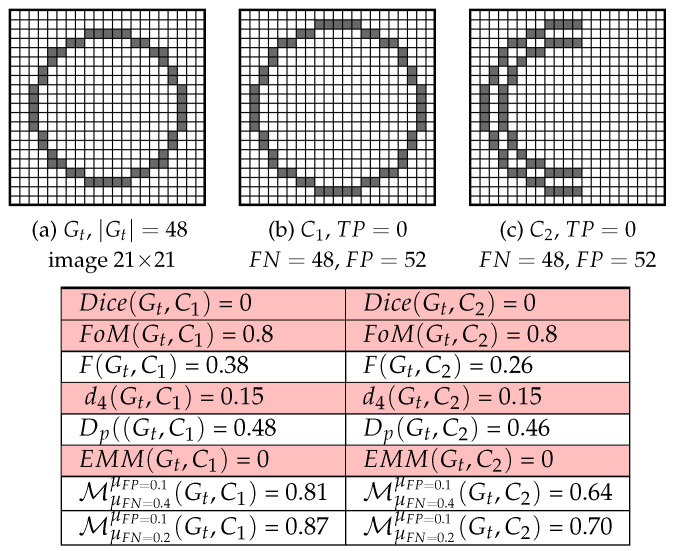
Different $D_{c}$: FPs and number of FNs are the same for $C_{1}$ and for $C_{2}$ (FN=48, FP=52), but the distances of FNs and the shapes of the two $D_{c}$ are different.

**Figure 3:**
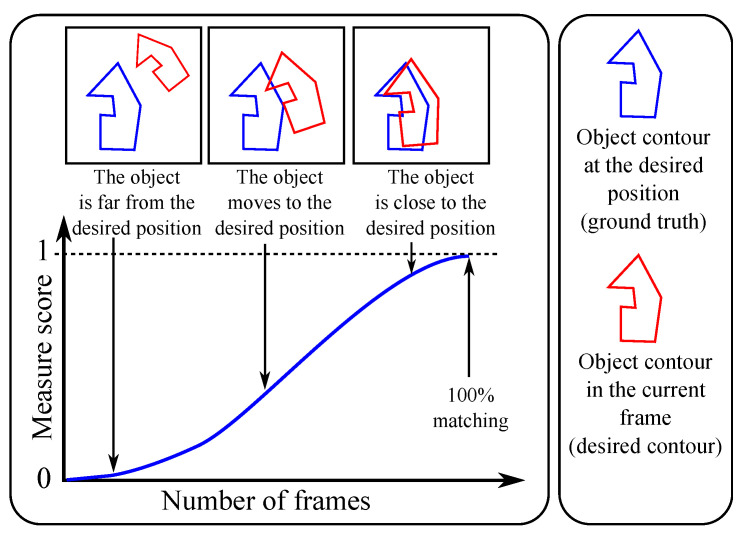
Expected behavior of a measure scores concerning an ideal displacement.

In reality, there are several alterations that can interfere with and disturb the object-pose estimation, including occlusion, translation, rotation or a change in the scale of the object. Consequently, both their own shape(s) and their contours may be changed. As an example, Figure 1 illustrates an object shape undergoing translation; due to discretization of the edges, shapes are not exactly similar. The purpose of this study is to determine when the object is moving to the desired position, or rather the opposite, moving away. To that end, six normalized supervised contour measures are presented below. Then, an evaluation process is performed to determine the degree to which an object shape differs from a desired position in function of various alterations. Various evaluation methods have been proposed in the literature to assess different shapes of edges using pixel-based ground truths (see reviews in [3,4,5,6,7]). Indeed, a supervised evaluation criterion calculates a measure of the dissimilarity between a ground truth ($G_{t}$) and a detected contour map ($D_{c}$) of an original image *I*, as in Figure 1 and Figure 2. In this paper, the closer the evaluation score is to 1, the more the object localization is qualified as appropriate, as represented in Figure 3. A score close to 0 indicates poor object positioning. The confusion matrix remains a cornerstone in evaluation methods for assessing a known shape. Comparing pixel by pixel $G_{t}$ and $D_{c}$, the first criterion assessed is the common presence of edge or non-edge points. A basic statistical evaluation is performed by combining $G_{t}$ and $D_{c}$. Subsequently, denoting |·| as the cardinality of a set (e.g., $\left|G_{t}\right|$ denotes the number of edge pixels in $G_{t}$), all points are categorized into four sets, as illustrated in Figure 1:

- True Positive points (TPs): $TP=\left|G_{t}\cap D_{c}\right|$,
- False Positive points (FPs): $FP=\left|\neg G_{t}\cap D_{c}\right|$,
- False Negative points (FNs): $FN=\left|G_{t}\cap \neg D_{c}\right|$,
- True Negative points (TNs): $TN=\left|\neg G_{t}\cap \neg D_{c}\right|$.

Various edge detection evaluation methods have been developed that make use of confusion matrices , cf. [5,6,7]. The Dice measure [8,9] is one well known example:

$$Dice\left(G_{t},D_{c}\right)=\frac{2\cdot TP}{2\cdot TP+FN+FP}.$$

This type of assessment is well suited to region segmentation evaluation [9], but one requirement for a reference-based edge map quality measure is the penalization of a displaced edge in function of FPs and/or FNs and also the distance from the correct position [6,7], as indicated with arrows in Figure 1.

In this context, Table 1 lists the most relevant normalized measures involving distances. For the pixel *p* in the candidate contour $D_{c}$, $d_{G_{t}}\left(p\right)$ represents the minimum Euclidian distance between *p* and $G_{t}$. Such distance measures are important in image matching and can be used to determine the resemblance between two object shapes [3]. To that end, if *p* belongs to $G_{t}$, $d_{D_{c}}\left(p\right)$ is the minimum distance between *p* and $D_{c}$, Figure 1 illustrates the difference between $d_{G_{t}}\left(p\right)$ and $d_{D_{c}}\left(p\right)$. Mathematically, denoting $\left(x_{p},y_{p}\right)$ and $\left(x_{t},y_{t}\right)$ the pixel coordinates of two points *p* and *t* respectively, thus $d_{G_{t}}\left(p\right)$ and $d_{D_{c}}\left(p\right)$ are described by:

$$\left\{\begin{array}{cc} \mathrm{for}\;p\in D_{c}:d_{G_{t}}\left(p\right) & =\mathrm{Inf}\left\{\sqrt{{\left(x_{p}-x_{t}\right)}^{2}+{\left(y_{p}-y_{t}\right)}^{2}},t\in G_{t}\right\},\;\\ \mathrm{for}\;p\in G_{t}:d_{D_{c}}\left(p\right) & =\mathrm{Inf}\left\{\sqrt{{\left(x_{p}-x_{t}\right)}^{2}+{\left(y_{p}-y_{t}\right)}^{2}},t\in D_{c}\right\}.\; \end{array}\right.$$

These distances are Euclidean, although certain authors include other types of distances, see [5,15,16]. For example, the Earth Mover’s Distance (EMD) represents a method to evaluate dissimilarity between two multi-dimensional distributions in some feature space using distance measures between single features. A distribution can be represented by a set of pixels [17]. This distance corresponds to the minimal cost to transform one distribution into the other. It is based on a solution to the transportation problem from linear optimization that minimizes the overall cost over all possible 1-to-1 correspondences. However, the main disadvantage of this technique appears when the two features contain several data that are too far away from each other, so EMD gives different weights for the points of the two sets, this optimization problem can be solved by partial matching [17]. Finally, EMD obtains a compactness of the matching signatures that can handle variable-size structures and can be computed quickly [16]. On the other and, the Chamfer distance expresses the computation of an average of the degree of matching, i.e., the average distance from each edge point to the nearest edge point in the ground truth template [18]. The advantage of this distance is that there is no necessity to use all the edge points of the shapes: for example, corner points or other feature points can be used. Nevertheless, the method lacks precision when too few feature points are taken into account and is sensitive to outliers, especially when the sample of data points is too light. For better robustness, as a compromise, the method works best when the point set is sparse, reducing the computation required.

In the field of shape positioning, other dissimilarity measures, based on the Hausdorff distance [3], have been proposed, see [3,4,6,19]. Most of these measures are non-normalized. The communication [6] proposes a normalization method for distance measures, but it is not sufficiently practical with real images. In the evaluation of edge detection, a commonly used normalized similarity measure refers to FoM [10]. Parameter κ acts as a scale parameter, the closer κ is to 1, the more FoM deals with FPs [6]. Nevertheless, FN distances are not recorded, and they are highly penalized as statistical measures (detailed in [7]):

$$FoM\left(G_{t},D_{c}\right)=\frac{1}{\max\left(\left|G_{t}\right|,\left|D_{c}\right|\right)}\;\cdot \;\left(TP+\sum_{p\in FP}\frac{1}{1+\kappa \cdot d_{G_{t}}^{2}\left(p\right)}\right).$$

Therefore, different shapes are interpreted as being the same [6] for the same number of FNs, as in Figure 2. Furthermore, if FP=0: $FoM\left(G_{t},D_{c}\right)=TP/|G_{t}|$. When FN>0 and $d_{G_{t}}^{2}\left(FP\right)$ is constant, it acts like matrix-based error assessments (detailed in [6]). Finally, for FP>0, FoM penalizes over-detection much less severely than under-detection [6]. Several evaluation measures have been derived from FoM: *F*, $d_{4}$, $D_{p}$ and EMM. First, contrary to FoM, the *F* measure calculates FN distances but not FP distances, so FPs are heavily penalized. However, the $d_{4}$ measurement is highly dependent on TP, FP, FN and ≈1/4 on FoM, but like the FoM measure $d_{4}$ penalizes FNs by around 25%. The right-hand term of the dissimilarity measure $D_{p}$ [13] calculates the distances of the FNs from the closest correctly detected edge pixel, i.e., TPs (FNs are heavily penalized when TPs are far from FPs, or when $G_{t}$∩$D_{c}=⌀$). In addition, $D_{p}$ has higher sensitivity to FNs than FPs because of the very high coefficient $\frac{1}{\left|I|-\right|G_{t}|}$ for the left-hand term (presented in detail in [7]). The Edge Mismatch Measure (EMM), on the other hand, depends on TPs and both $d_{D_{c}}$ and $d_{G_{t}}$. Thus, $\delta_{D_{c}/G_{t}}\left(p\right)$ is a threshold distance function that penalizes distances exceeding a maximum value maxdist). It should be noted that the parameters suggested depend on |I|, the total number of pixels in *I*. Moreover, EMM only calculates a score other than 0 if there is at least one TP, see example in Figure 2 with two different shapes, but obtaining the same scores.

## 3. A New Normalized Measure

The principal motivation is that currently there is no normalized shape-based measure that takes into account both FP and FN distances and can record a desired evolution in the localization of the object. As explained in [20], FP and FN distance evaluations must not be symmetrical. Evidently, a shape-based measure involving false negative distances is more accurate than other techniques. However, using only undersegmentation measures, where parts of the candidate image are missing but detected near their desired positions, they are not taken into account (by *F* for example, see Table 1) and the object is poorly localized. Missing edges need to be more heavily penalized than spurious edges because isolated points can disturb the shape localization, and therefore most of the measures, cf. experiments. To summarize, a measure needs to penalize FNs more highly than FPs, because the more FNs there are in $D_{c}$, the more the shape of the desirable object is difficult to unrecognize and therefore difficult to localize.

Thus, in separating penalties for FN distances and FP distances, the new normalized distance measure is inspired by the *Relative Distance Error* [3,7,21,22]:

$$\mathrm{RDE}\left(G_{t},D_{c}\right)=\sqrt{\frac{1}{\left|D_{c}\right|}\cdot \sum_{p\in D_{c}}d_{G_{t}}^{2}\left(p\right)}+\sqrt{\frac{1}{\left|G_{t}\right|}\cdot \sum_{p\in G_{t}}d_{D_{c}}^{2}\left(p\right)}.$$

**Figure 4:**
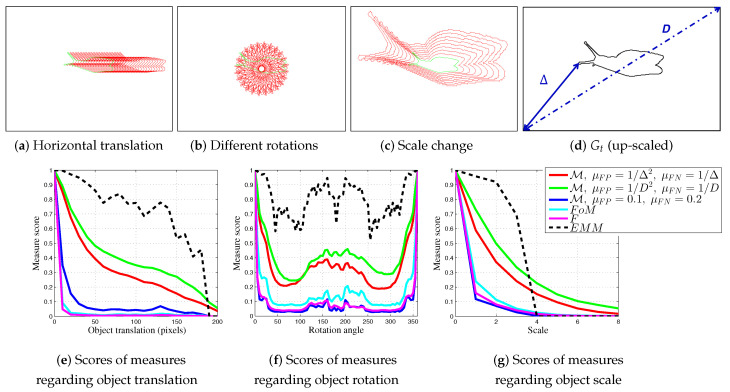
Examples behaviors of localization metrics for translation, rotation, and scale alterations. In (**a**–**c**), red points represent the shape at a particular position, whereas the green points correspond to the true shape position (i.e., $G_{t}$). Several parameters for M are tested: Δ represents the maximum distance between a pixel in $D_{c}$ with $G_{t}$ (usually an image corner pixel), whereas *D* is the length of the image diagonal. Parameters *D* and Δ are calculated automatically and D>Δ.

Indeed, this edge detection evaluation measure separately computes the distances of FPs and FNs in function of the number of points in $D_{c}$ and $G_{t}$, respectively, but it is not normalized; so its scores are interpretable with difficulty (Appendix A of this paper presents other non-normalized measures with results regarding real videos V2, V3 and V4.). Thereafter, demonstrations and experiments in [7,20] provide the motivations for the elaboration of a normalized shape-based location described by the following formula, when FN>0 or FP>0:

(1) $$M\;\left(G_{t},D_{c}\right)=\frac{1}{FP+FN}\cdot \;\left[\frac{FP}{|D_{c}|}\cdot \;\;\sum_{p\in D_{c}}\frac{1}{1+\mu_{FP}\cdot d_{G_{t}}^{2}\left(p\right)}+\frac{FN}{|G_{t}|}\cdot \;\;\sum_{p\in G_{t}}\frac{1}{1+\mu_{FN}\cdot d_{D_{c}}^{2}\left(p\right)}\right],$$

where $\left(\mu_{FP},\mu_{FN}\right)$ are real positives representing the two scale parameters and the coefficient $\frac{1}{FP\;+\;FN}$ normalizes the M function. If FP=FN=0, then M=1. Subsequently, to become as fair as possible, FPs and FNs distances are penalized separately according to the relationship between FPs and $|D_{c}|$ and between FNs and $|G_{t}|$ respectively, ensuring an equal distribution of mistakes, without symmetry of penalties. The two parameters $\mu_{FP}$ and $\mu_{FN}$ tune the evaluation respectively for FPs and FNs. Indeed, when $\mu_{FP}<\mu_{FN}$, M penalizes the FNs more, compared to the FPs, as illustrated in Figure 2. The results presented below show the importance of the weights given for FNs because isolated FP points may disturb the shape localization. In this context, Section 4.3 underlines that the optimum values for the parameters $\left(\mu_{FP},\mu_{FN}\right)$ should be linked to the maximum Euclidian distance between $G_{t}$ and any pixel in the image (see Δ parameter in Figure 4d).

## 4. Evaluation and Results

To test various parameters and check whether the proposed measure has the required properties, several alterations are made to create synthetic localization results simulating real results. To quantify the reliability of a measure of dissimilarity, various alterations are applied to an edge map of a synthetic shape: rotation, translation and scale change (in Figure 4). This verifies whether the evolution of the score obtained by a measure corresponds with the expected behavior: usually minor errors for close shapes (scores close to 1) and heavier penalties for more different shapes (scores close to 0), as illustrated in Figure 3. To summarize, the desired behavior of a normalized dissimilarity measure is that its score should:

- increase towards 1 when the shape approaches its target,
- converge slowly towards 1 when the movement towards the target is slow,
- rise rapidly towards 1 when the movement towards the target is rapid,
- not be disturbed (error peaks, see results in Appendix A) by the sudden appearance of outliers or the disappearance of some feature pixels,
- remain stable (i.e., constant) when the object is immobile, despite the undesirable contours (outliers) detected during the video.

The next step consists of experiments carried out concerning real videos, by computing contours.

### 4.1. Experiments with Synthetic Shapes

A synthetic shape is created and presented in Figure 4d. This image is inverted for a better visualization, i.e., edge points tied to the object are in black whereas background and non-shape points are represented in white. In Figure 4a,b, red pixels correspond to the shape of the desired object of a simulated movement and green pixels represent the object shape at the desired position (exactly positioned as the ground truth $G_{t}$).

#### 4.1.1. Translation

In the first test, the synthetic contour shape is gradually translated by moving it away from its initial location along a horizontal straight line. Figure 4a illustrates this movement and Figure 4e reports the values of FoM, *F*, EMM and M. The new algorithm is tested with different parameters $\left(\mu_{FP},\mu_{FN}\right)$, considering 1/D or 1/Δ. Thus, *D* is the diagonal length of the image. Δ is the maximum distance between a pixel in $D_{c}$ with $G_{t}$ (usually an image corner pixel), as illustrated in Figure 4d. Three couples of parameters are tested : ($\mu_{FP}=1/\Delta^{2}$, $\mu_{FN}=1/\Delta$), ($\mu_{FP}=1/D^{2}$, $\mu_{FN}=1/D$) and ($\mu_{FP}=0.1$, $\mu_{FN}=0.2$). They are chosen such that $\mu_{FP}<\mu_{FN}$ to penalize FNs more highly than FPs. The Dice and $d_{4}$ scores are not reported because they have clear discontinuities and are highly sensitive to small displacements (see [20]). The FoM and *F* measures are also highly sensitive to small displacements, as M with $\mu_{FP}=0.1$ and $\mu_{FN}=0.2$; moreover, as with EMM, they are non-monotonous (unlike M with automatic parameters tied to *D* and Δ). This first experiment shows the importance of parameter choice concerning $\left(\mu_{FP},\mu_{FN}\right)$; they must be far below 0.1.

#### 4.1.2. Rotation

The second test is performed by incrementally rotating the control shape until complete $360^{\circ }$ rotation, as illustrated in Figure 4b. The shape of the measure scores curve should be roughly symmetrical at around 180${}^{\circ }$. The FoM and *F* measures are highly sensitive to small rotations and EMM does not sufficiently penalize movements, whereas M, considering Δ or *D* parameters, results in consistent scores. Indeed, the scores are between 0.3 and 0.5 because edges of $D_{c}$ are always located in the same neighborhood as edges of $G_{t}$, contrary to other measures where the scores are less than 0.2.

#### 4.1.3. Scale Change

The last experiment on synthetic data involves scaling up the object shape with the maximum scale 8 times the original (nevertheless, $G_{t}$ and $D_{c}$ keep the same size). However, the EMM curve has sharp discontinuities showing its unstable response to scaling, because its responses depend strongly on the number of TPs and correspond to 0 without TPs. If there is no TP, for bigger scales, EMM falls to 0, with no evolution in the score for up-scaling. The FoM and *F* scores become very sensitive right from the first change with scores close to 0.2. Finally, M with automatic parameters Δ or *D* obtains desirable scores, decreasing regularly and monotonously from 1 to 0.

### 4.2. Experiments on Real Images

**Figure 5:**
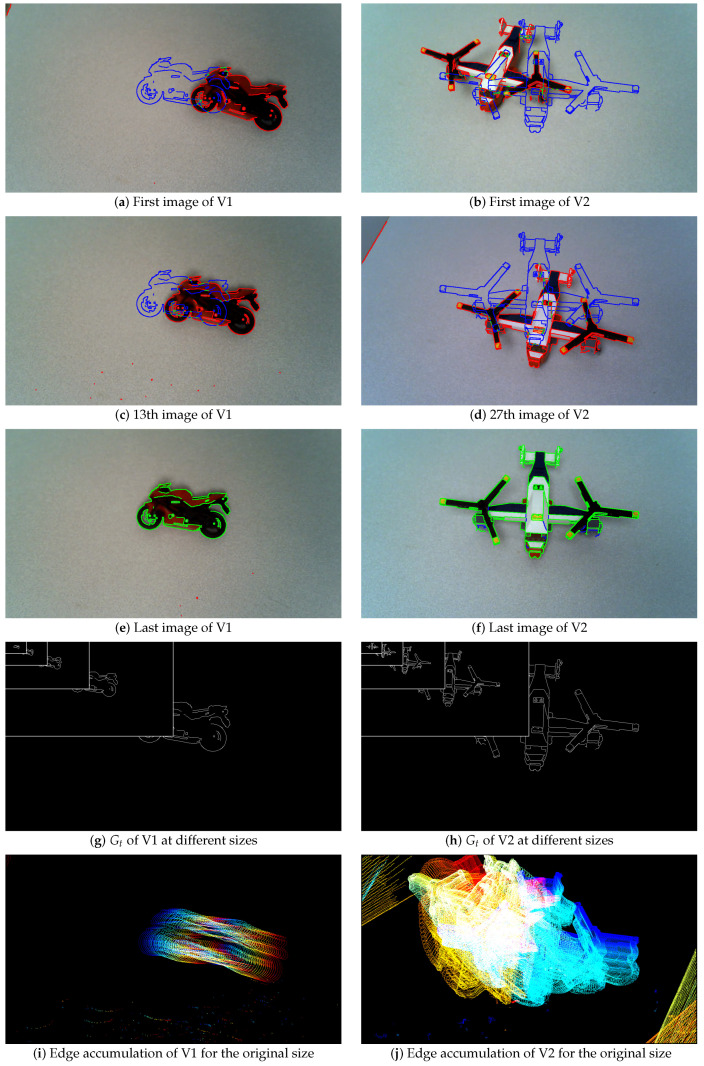
The first images of videos V1 and V2 with their $G_{t}$ for different sizes: original size (1280 × 720), 4×, 16×, 64× and 256× reduced (640 × 360, 320 × 180, 160 × 90 and 80 × 45).

**Figure 6:**
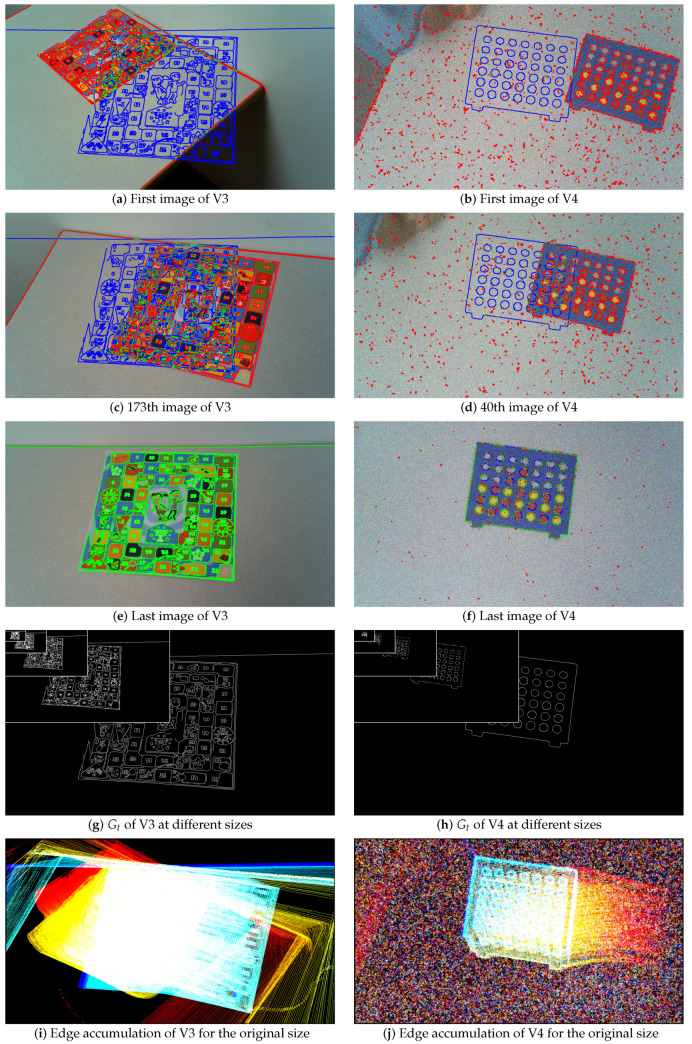
The first images of videos V3 and V4 with their $G_{t}$ at different sizes: original size (1280 × 720), 4×, 16×, 64× and 256× reduced (640 × 360, 320 × 180, 160 × 90 and 80 × 45).

**Figure 7:**
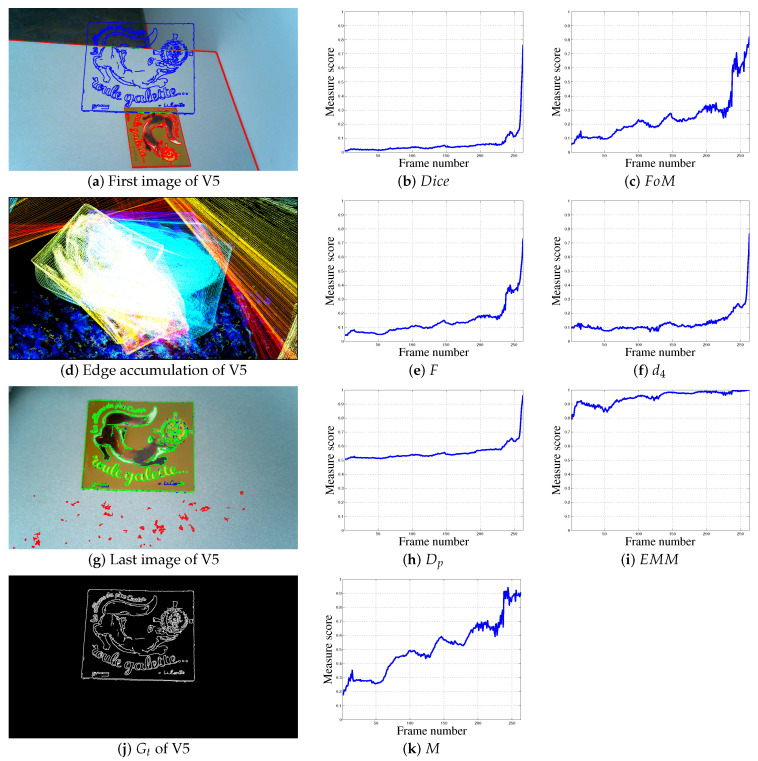
Localization metrics behaviors in a real experiment concerning video V5, 264 frames, with images of size 720 × 1280.

**Figure 8:**
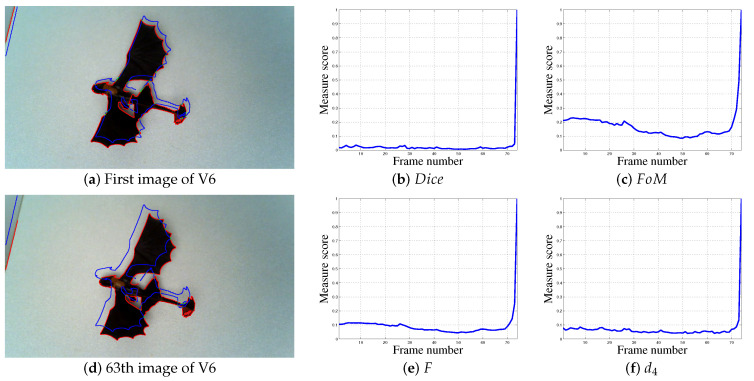

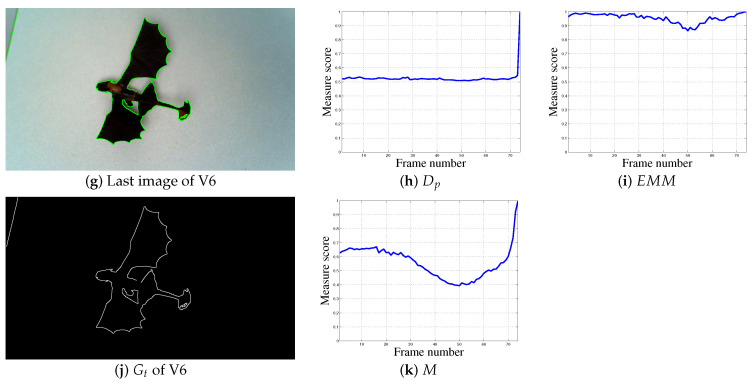
Localization metrics behaviors for a real experiment concerning video V6, 74 frames, with images of size 720 × 1280.

Experiments on real color images are also carried out, see Figure 5, Figure 6, Figure 7 and Figure 8 and Table 2. The Canny edge detector (σ=1) [23] is used to extract thin edges. Figure 5 and Figure 6 illustrate the edge detection, compared to the ground truth. The edge detections are shown on images at the original size 1280 × 720, whereas the ground truths are presented in Figure 5g,h and Figure 6g,h at different scales in the same image. Figure 7 and Figure 8 presents two other experiments with images at one size 1280 × 720. The aim, by moving the camera and using thin binary edges as features, is to determine when the object is in the desired position in the image. The scores must converge to 1. The desired position corresponds to the object in the last video frame (usually blue edges). The ground truth corresponds to the binary boundaries of the desired position of the known object, represented by blue pixels in Figure 5a–f, Figure 6a–f, Figure 7a–g and Figure 8a,d,g. The green pixels represent TPs, red points are FPs, whereas blue pixels, which are also $G_{t}$, are FNs. These features are dilated using a structural element of size 3 × 3 for better visualization; after which they are finally inserted into the current frame. During the movement, each frame may become corrupted by numerous FPs. Moreover, the candidate object may contain FNs when the object is well positioned, as illustrated in Figure 11e,f. The images presented in Figure 5i,j, Figure 6i,j and Figure 7d represent the edge movements in function of time (from blue to red), illustrating the huge number of noise pixels for certain videos. Please note that FoM, *F*, $d_{4}$, $D_{p}$ and EMM measures are compared using default parameters (see Table 1).

#### 4.2.1. Real Video 1 (V1)

**Figure 9:**
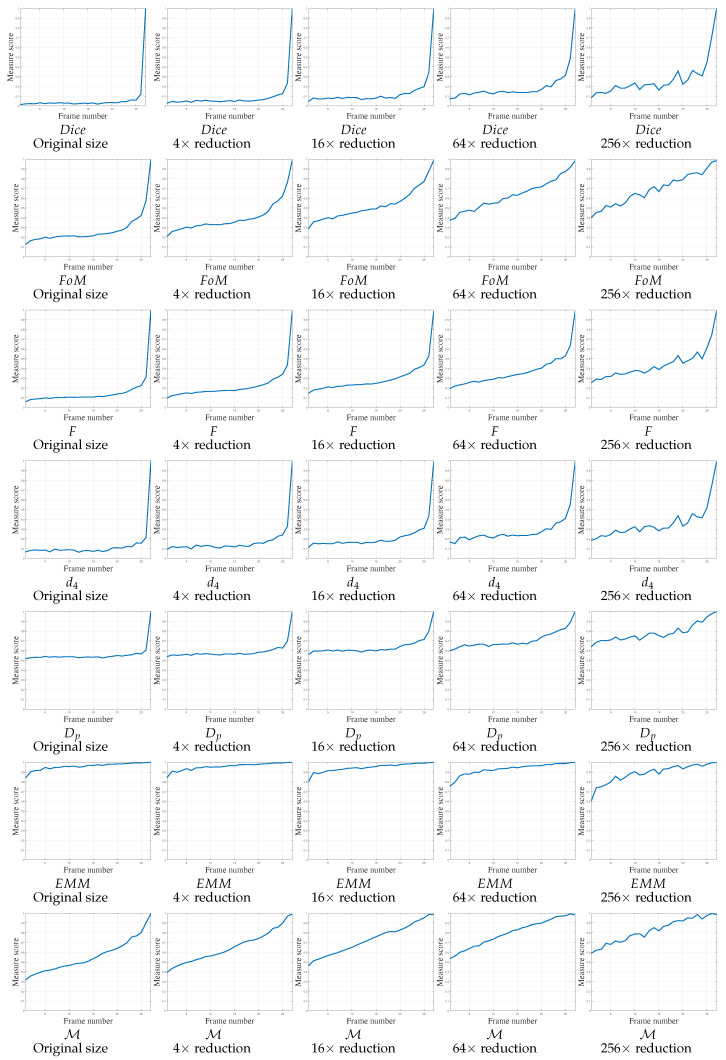
Localization metrics behaviors on real experiment concerning video 1 (V1) of 27 frames. The parameter concerning FoM, *F*, $d_{4}$ and $D_{p}$ is κ=1/9. Concerning M, the parameters are $\mu_{FP}$ = $1/\Delta^{2}$ and $\mu_{FN}=1/\Delta$, so $\mu_{FP}<\mu_{FN}$.

The first video, presented in Figure 5 (left), contains 27 frames. This pose evaluation predominantly concerns translation; some undesirable FPs are also present and may disturb the object position assessment. Object contours are easily extracted throughout the video. The scores of the various measures are reported in Figure 9 in function of image size. The object is always visible in the image throughout the whole video. As this experiment only relates to a regular object translation, the score of the measures must start around 0.5, increasing regularly and monotonously up to 1 for each scale. For large scales, Dice, FoM, *F*, $d_{4}$ and $D_{p}$ increase to 1 exclusively around the last frames. FoM has correct behavior for the two smallest scales (160 × 90 and 80 × 45). On the contrary, EMM scores are close to 1 from the beginning of the video. Only M obtains desirable behavior, increasing regularly and monotonously up to 1, in accordance with each scale of the images.

#### 4.2.2. Real Video 2 (V2)

**Figure 10:**
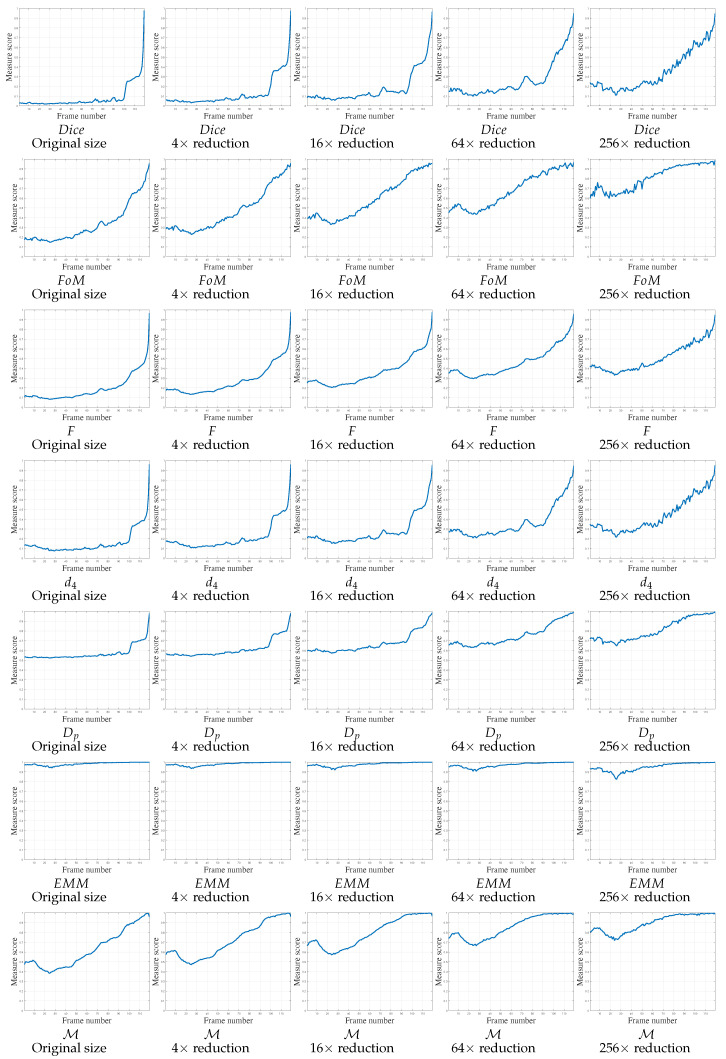
Localization metrics behaviors on real experiment concerning video 2 (V2) of 119 frames. The parameter concerning FoM, *F*, $d_{4}$ and $D_{p}$ is κ=1/9. Concerning M, the parameters are $\mu_{FP}$ = $1/\Delta^{2}$ and $\mu_{FN}=1/\Delta$, so $\mu_{FP}<\mu_{FN}$.

Regarding the second video, V2, a rotation and a small translation are imposed on the camera, as can be observed in Figure 5b,d,f . Figure 5j illustrates the object rotation. These movements create a slight scale change of the object. Moreover, the table borders create FPs at several moments. The object is moving to its desired location for the 10 first frames, then it is moving beyond the desired position, as shown in Figure 5d. Thereafter, it moves smoothly to its desired position, with the desired shape superposing $G_{t}$. The scores of the various measures are reported in Figure 10 in function of image size. Most of the measures do not detect when the object moves beyond the desired position after 10 frames. Concerning Dice, $d_{4}$ and $D_{p}$, the scores converge to 1 for the last frames. FoM and *F* measures do not sufficiently mark the cavity in the curve after 10 frames, except for small images. Also, the scores tied to EMM are too close to 1, which are not exploitable. Finally, the scores of the proposed measure M mark the cavity in the curve after 10 frames for each image scales, and then converging to 1, when the object arrives in the desired position.

#### 4.2.3. Real Video 3 (V3)

**Figure 11:**
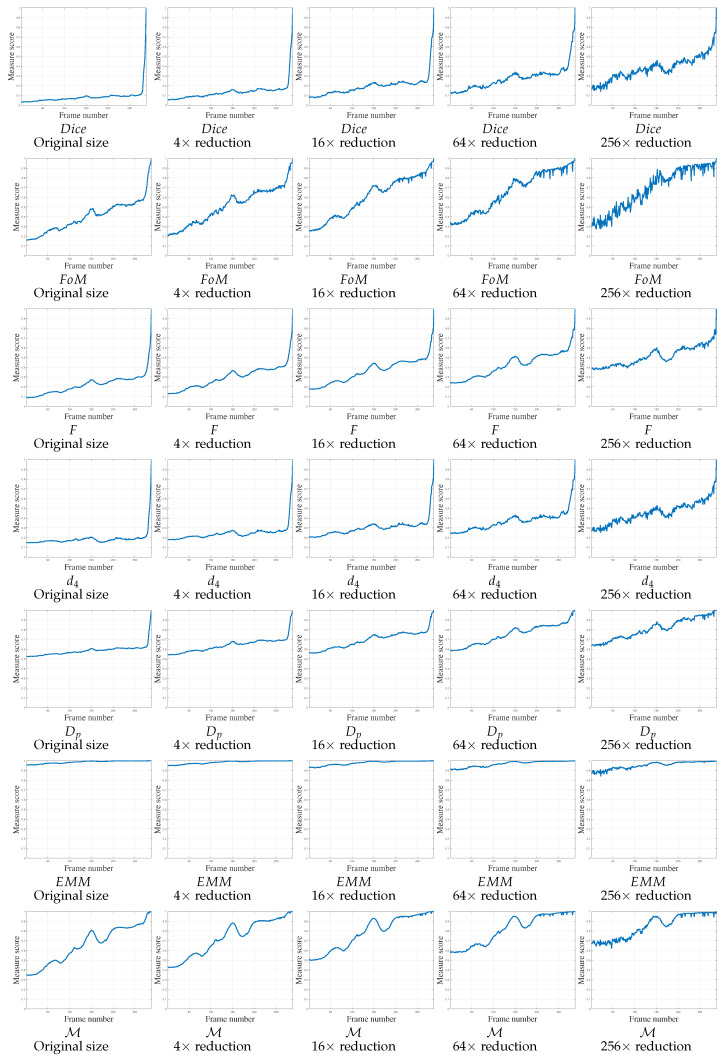
Localization metrics behaviors on real experiment concerning video 3 (V3) of 289 frames. The parameter concerning FoM, *F*, $d_{4}$ and $D_{p}$ is κ=1/9. Concerning M, the parameters are $\mu_{FP}$ = $1/\Delta^{2}$ and $\mu_{FN}=1/\Delta$, so $\mu_{FP}<\mu_{FN}$.

For the third video, V3, the object contours are extracted easily, with false positive undesirable points created by the table edge (Figure 6a,c) and camera rotation. This camera rotation changes the scale of the candidate shape, which may adversely affect contour-based localization. The object moves to its desired position, up to 150 frames (creating a bump curve), then moves away and then returns to its final position, superimposing $G_{t}$. The scores of the various measures are reported in Figure 11 in function of image size. The EMM and $D_{p}$ curves are not significant because the movement is not really perceived by the measures. In addition, the Dice, *F* and $d_{4}$ scores only converge to 1 when the candidate object is close to the desired location for large image scales. Only the FoM and M measures exhibit the intended behavior for this video sequence, even if the FoM scores for small images are globally noisy.

#### 4.2.4. Real Video 4 (V4)

**Figure 12:**
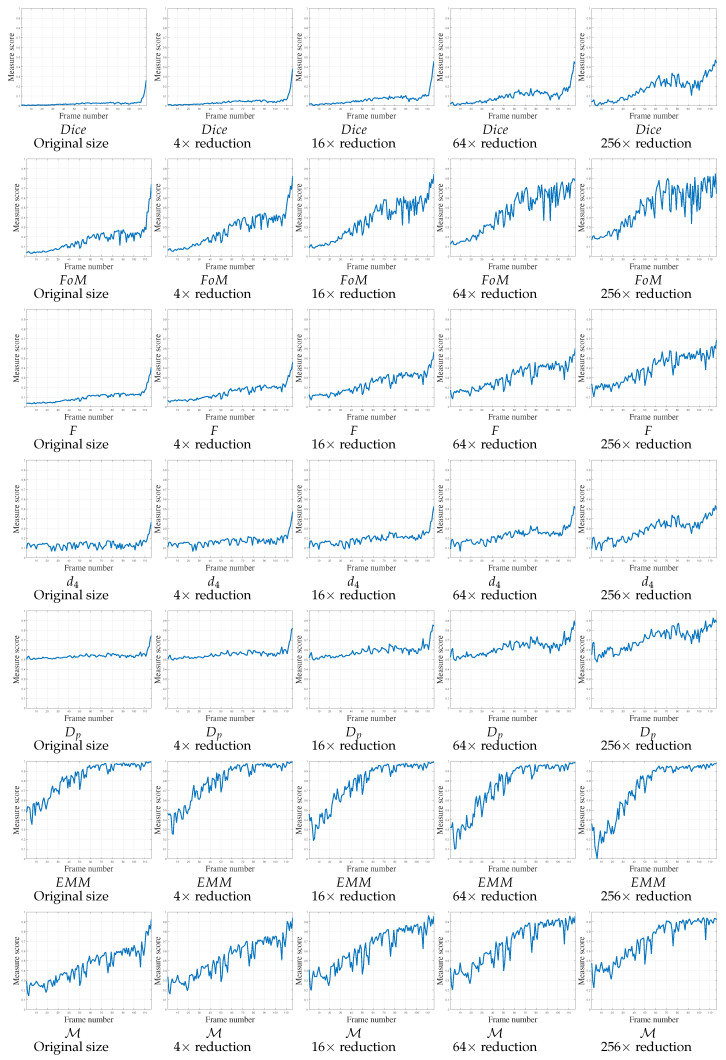
Localization metrics behaviors on real experiment concerning video 4 (V4) of 116 frames. The parameter concerning FoM, *F*, $d_{4}$ and $D_{p}$ is κ=1/9. Concerning M, the parameters are $\mu_{FP}$ = $1/\Delta^{2}$ and $\mu_{FN}=1/\Delta$, so $\mu_{FP}<\mu_{FN}$.

**Figure A3:**
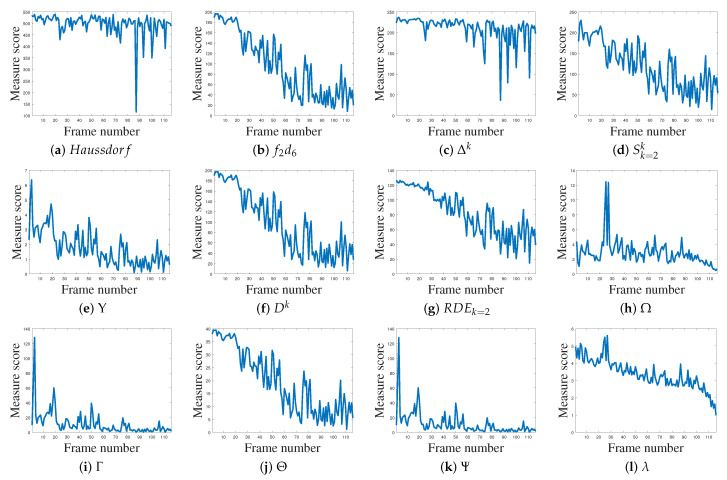
Behaviors of non-normalized localization metrics on real experiment with noisy images tied to video 4 (V4), with images of size 720 × 1280, see Figure 6. Plots from [20].

The fourth video, V4, is severely corrupted by random noise on each color plane (SNR ≈ 11 dB). These disturbances create spurious pixels in the edge detection process, but more particularly, the candidate object edges are not well localized or even absent. Therefore, in Figure 12, most measures do not evolve monotonously, but constantly for each image size, except for the end of the video, as Dice, $d_{4}$ and $D_{p}$. The scores for the *F* measure increase but do not converge to 1 at the end of the movement, they increase until around 0.5, like the final scores of Dice and $d_{4}$. On the contrary, $D_{p}$ scores start around 0.5 and remain constant around this value up to the last frames (except for the smallest resolution). The FoM scores increase at the end of the video, but are stochastic for small videos, with a gap of up to 0.4 between two frames. The EMM measure converges rapidly, but remains constant until the end. Finally, the M measure increases monotonously to 1 in accordance with the different resolutions. The gaps do not disturb the usual shape of the curves, with a score converging to 1. A comparison with other curves regarding non-normalized measures are presented in Appendix A, Figure A3.

#### 4.2.5. Real Video 5 (V5)

The results given in Figure 7 and Figure 8 are only at one scale (i.e., 720 × 1280). Video V5 contains 264 frames. The shape of the object undergoes considerable translation, rotation, and scale-change. Figure 7d shows the various movements of the object that occur in the video. It should be noted that the noise pixels caused by the texture of the table are also present in images $D_{c}$, which could disturb the localization. During the video, the object moves in a series of steps (with pauses) towards its desired position. These steps appear clearly with measure M, but less so with FoM and even less with *F*. The Dice, $d_{4}$ and $D_{p}$ scores are somewhat constant, only changing significantly at the end of the video. Measure EMM remains close to 1 and it can be assumed that the object in in its desired position after about 150 frames.

#### 4.2.6. Real Video 6 (V6)

The last video, V6, contains 74 frames. The contours of the object are well detected and there are no noise pixels. The object is very close to its final position, so the scores of a normalized measure should be higher than 0.5 at the start of the video. Until midway through the video, the object undergoes a constant translation without particularly moving towards or away from its desired position. However, after some 20 frames, the object moves away from its target before returning to the right position. The Dice and $d_{4}$ scores stay close to 0 almost throughout the video before jumping directly to 1 for the last frame. The scores for measure $D_{p}$ remain relatively constant around 0.5 before jumping to 1. Although the appearance of the FoM and *F* curves show that the object moves away from its target after 20 frames, the scores are too close to 0 at the start of the video and converge too quickly to 1 in the final frames. The M measure, on the other hand, behaves in the desired way: a score above 0.5 at the start of the video which then decreases after about 20 frames before converging steadily to 1 after 50 frames. This result shows exactly the desired behavior of a normalized shape similarity measure.

### 4.3. Influence of the Parameters

**Figure 13:**
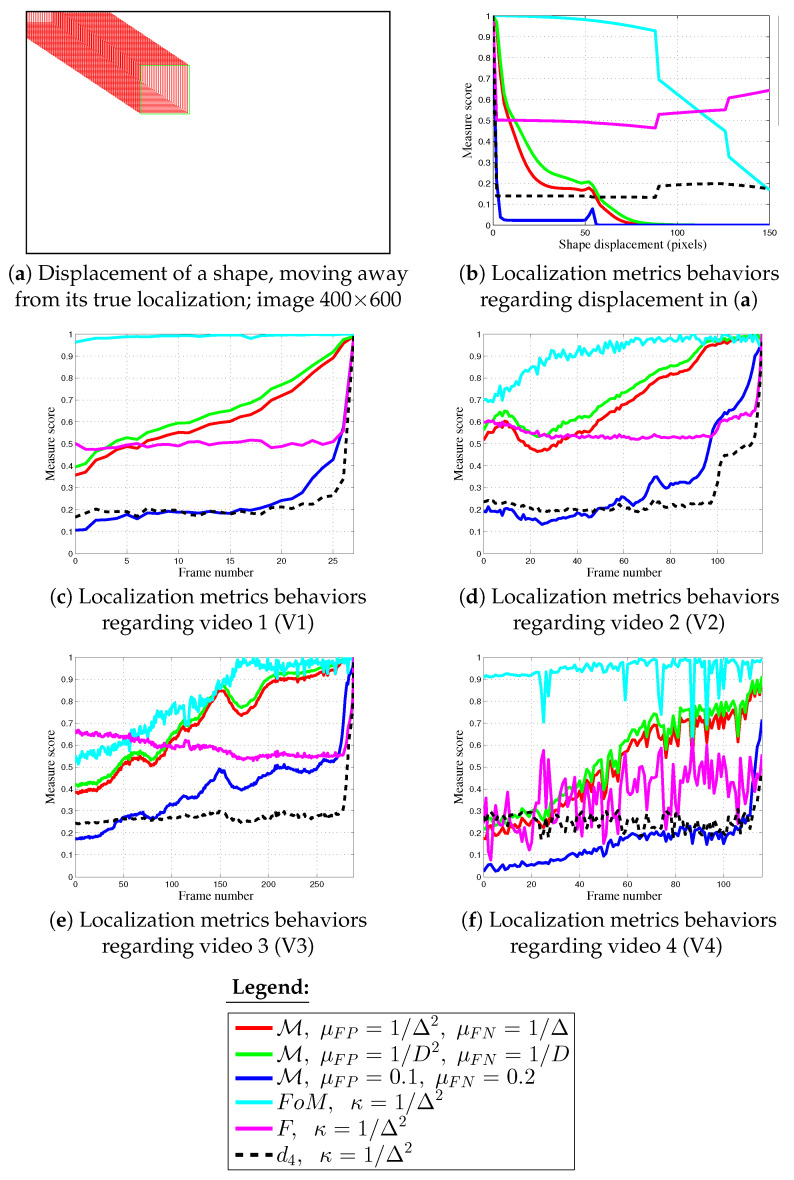
Comparison of score evolution regarding synthetic and real videos 4× reduced with $\kappa =1/\Delta^{2}$ for FoM, *F* and $d_{4}$ shape measures. Different parameters tied to M: $\mu_{FP}$ and $\mu_{FN}$ are also tested.

The last experiments presented in Figure 13 show the importance of parameter choice, these complete the previous experiments available in Section 4.1 and in Figure 4. To supplement the tests, the FoM, *F* and $d_{4}$ measures are compared using $\kappa =\frac{1}{\Delta^{2}}$, which is similar to M parameters during the previous experiments. The curves presented in Figure 13b–f illustrate that such a value is completely inappropriate for these shape detection approaches. First, the experiment in Figure 13a concerns a synthetic shape which is moving away from its true location. M, when, $\mu_{FP}=0.1$ and $\mu_{FN}=0.2$ decreases until 0 too rapidly whereas, using other parameters, it behaves correctly as a function of the shape displacement. Other normalized shape dissimilarity measures with $\kappa =\frac{1}{\Delta^{2}}$ create important gaps in their plotted scores. Moreover, *F* and $d_{4}$ are not monotonous. This gap is created when the shape is moving outside of the image; so numerous points of $D_{c}$ are disappearing.

Regarding real videos, FoM scores remain close to 1 throughout the videos or converge rapidly to 1, as for V3. Also, *F* decreases using this parameter for V2 et V3 (apart from the final frames), which is in opposition to the assessment being sought here. The scores tied to *F* and V1 are also constant around 0.5, whereas they are very stochastic concerning V4. On the contrary, plotted scores tied to $d_{4}$ are similar to scores in Figure 9, Figure 10, Figure 11 and Figure 12 when κ=1/9. These results have a natural flow because $d_{4}$ is composed of 3/4 of statistics (number of FPs, FNs, and TPs). Concerning M, when, $\mu_{FP}=0.1$ and $\mu_{FN}=0.2$, it behaves as FoM when κ=1/9 ( see Figure 9, Figure 10, Figure 11 and Figure 12). Finally, the use of $\mu_{FP}=\frac{1}{\Delta^{2}}$ and $\mu_{FN}=\frac{1}{\Delta }$ parameters obtains $\mu_{FP}<\mu_{FN}$ for each scale, penalizing more heavily FNs compared to FPs in Equation (1), as demonstrated in [20]. Thus, instead of *D* the choice of Δ is preferable when it comes to certain shapes. Moreover, when $\mu_{FP}=\frac{1}{D^{2}}$ and $\mu_{FN}=\frac{1}{D}$, scores of M converge too rapidly to 1, justifying the choices for its parameters.

## 5. Conclusions

A new approach to measuring a contour-based object pose is presented in this paper. The new algorithm enables supervised assessment of the recognition and localization of known objects as a function of false positive (FP) and false negative (FN) distances. The two parameters $\mu_{FP}$ and $\mu_{FN}$ tune the evaluation respectively for FPs and FNs. When $\mu_{FP}<\mu_{FN}$, the proposed approach M penalizes FNs more heavily than FPs. This allows the use of efficient weights for FNs because isolated FPs could disturb the shape localization without this condition. The results of several experiments carried out on synthetic images are presented alongside the results of the current best shape-based normalized algorithms to show the comparative strength of the innovative method. Also, experiments on real images showcase the pertinence of the approach for estimating object pose or shape-matching. The new measure is normalized, which is a major advantage for qualifying the position of an object shape. In addition, it can be used on smaller-sized images than other measures, with a corresponding gain in processing times. Tests on images at several scales show the reliability of M, because the shapes of the curves are similar, with no large gaps between each scale. Moreover, the new normalized localization assessment does not need any tuning parameters because $\mu_{FP}$ and $\mu_{FN}$ are computed automatically with the ground truth ( the shape of the object at the ideal positioning). Finally, this localization measure may be useful for visual servoing processes or loss function in machine learning. Future work will consist of a deeper investigation by evaluating the combination of reducing images and the Chamfer distance for the shape-matching process. 

## Figures and Tables

**Table 1:** List of normalized dissimilarity measures involving distances, generally: κ=0.1 or 1/9.

Error Measure Name	Formulation	Parameters
Pratt’s Figure of Merit [10]	$FoM\left(G_{t},D_{c}\right)=\frac{1}{\max\left(\left|G_{t}\right|,\left|D_{c}\right|\right)}\cdot \sum_{p\in D_{c}}\frac{1}{1+\kappa \cdot d_{G_{t}}^{2}\left(p\right)}$	$\kappa \in ]0;1]$
FoM revisited [11]	$F\left(G_{t},D_{c}\right)=\frac{1}{\left|G_{t}\right|+\beta \cdot FP}\cdot \sum_{p\in G_{t}}\frac{1}{1+\kappa \cdot d_{D_{c}}^{2}\left(p\right)}$	$\kappa \in ]0;1]$ and β a real positive
Combination of FoM and statistics [12]	$d_{4}\left(G_{t},D_{c}\right)=1-\frac{1}{2}\cdot \sqrt{\frac{{\left(TP-\max\left(\left|G_{t}\right|,\left|D_{c}\right|\right)\right)}^{2}+FN^{2}+FP^{2}}{{\left(\max\left(\left|G_{t}\right|,\left|D_{c}\right|\right)\right)}^{2}}+{\left(1-FoM\left(G_{t},D_{c}\right)\right)}^{2}}$	$\kappa \in ]0;1]$
Edge map quality measure [13]	$D_{p}\left(G_{t},D_{c}\right)=1-\frac{1/2}{\left|I\right|-\left|G_{t}\right|}\;\cdot \;\sum_{p\in FP}\;\;\left(1-\frac{1}{1+\kappa \;\cdot \;d_{G_{t}}^{2}\;\left(p\right)}\right)-\frac{1/2}{\left|G_{t}\right|}\;\cdot \;\sum_{p\in FN}\;\;\left(1-\frac{1}{1+\kappa \;\cdot \;d_{TP}^{2}\;\left(p\right)}\right)$	$\kappa \in ]0;1]$
Edge Mismatch Measure (EMM) [14]	$EMM\left(G_{t},D_{c}\right)=\frac{TP}{TP+\omega \cdot \left[\sum_{p\in FN}\delta_{D_{c}}\left(p\right)+\epsilon \cdot \sum_{p\in FP}\delta_{G_{t}}\left(p\right)\right]}$	$M_{dist}$, $D_{max}$, ω and ϵ are real positive.
	$\delta_{D_{c}}\left(p\right)=\left\{\begin{array}{cc} d_{D_{c}}\left(p\right), & \mathrm{if}\;d_{D_{c}}\left(p\right)<M_{dist}\\ D_{max}, & \mathrm{otherwise} \end{array}\right.\;\mathrm{and}\;\delta_{G_{t}}\left(p\right)=\left\{\begin{array}{cc} d_{G_{t}}\left(p\right), & \mathrm{if}\;d_{G_{t}}\left(p\right)<M_{dist}\\ D_{max}, & \mathrm{otherwise}. \end{array}\right.$	$M_{dist}$ = |I|/40, $D_{max}=\left|I\right|/10$, ω=10/|I|, ϵ=2, see [14].

**Table 2:** Summary of different alterations imposed for each video. The degree of noise signifies the number of FPs outside of the shape contour of the desired object (due to noise or a table border). The number of “*” corresponds to the degree of noise, object translation, rotation or scale change; the more the stars “*” are, the more the image is altered.

	V1	V2	V3	V4	V5	V6
Degree of noise	*	**	**	***	**	-
Degree of Translation	***	*	**	**	***	*
Degree of Rotation	-	**	***	-	***	-
Degree of object scale change	-	*	**	*	**	-

**Figure A1:**
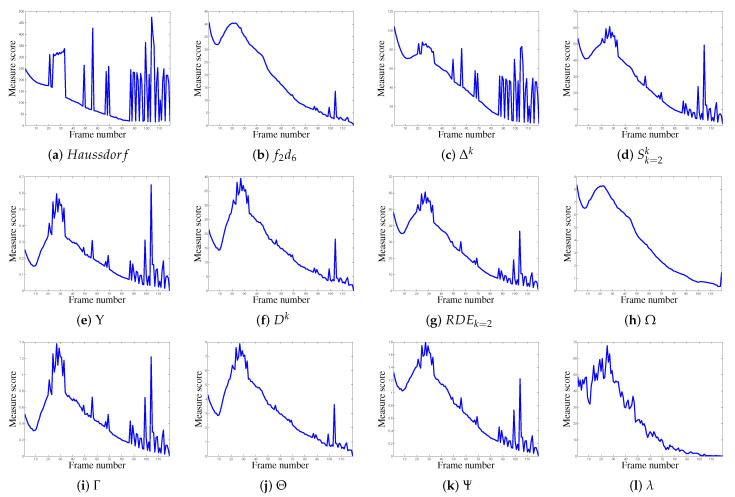
Behaviors of non-normalized localization metrics on real experiment with noisy images tied to video 2 (V2), with images of size 720 × 1280, see Figure 5.

**Table:** 

			
(**a**) Haussdorf	(**b**) $f_{2}d_{6}$	(**c**) $\Delta^{k}$	(**d**) $S_{k=2}^{k}$
			
(**e**) Y	(**f**) $D^{k}$	(**g**) $RDE_{k=2}$	(**h**) Ω
			
(**i**) Γ	(**j**) Θ	(**k**) Ψ	(**l**) λ

**Figure A2:**
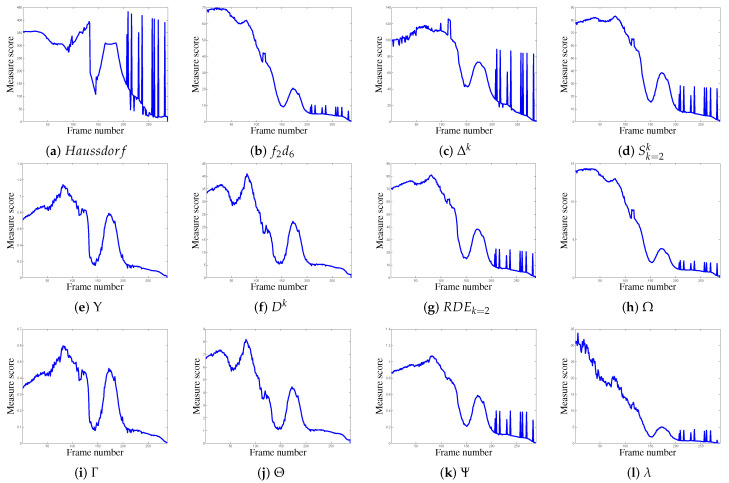
Behaviors of non-normalized localization metrics on real experiment with noisy images tied to video 3 (V3), with images of size 720 × 1280, see Figure 6.

**Table:** 

			
(**a**) Haussdorf	(**b**) $f_{2}d_{6}$	(**c**) $\Delta^{k}$	(**d**) $S_{k=2}^{k}$
			
(**e**) Y	(**f**) $D^{k}$	(**g**) $RDE_{k=2}$	(**h**) Ω
			
(**i**) Γ	(**j**) Θ	(**k**) Ψ	(**l**) λ

**Table:** 

			
(**a**) Haussdorf	(**b**) $f_{2}d_{6}$	(**c**) $\Delta^{k}$	(**d**) $S_{k=2}^{k}$
			
(**e**) Y	(**f**) $D^{k}$	(**g**) $RDE_{k=2}$	(**h**) Ω
			
(**i**) Γ	(**j**) Θ	(**k**) Ψ	(**l**) λ

## Appendix A

Appendix A presents additional results on real images, regarding videos 2 (V2), 3 (V3) and 4 (V4), presented respectively in Figure 5 and Figure 6, with images of size 720 × 1280. The results presented here enable us to compare the behaviors of the measures presented in this paper with those obtained using non-normalized measures. The non-normalized measures are mathematically defined in Table A1. They have been detailed and tested in [7]. Please note that the MATLAB code of FoM, $D^{k}$, $S^{k}$ and $\Delta^{k}$ measures are available at http://kermitimagetoolkit.net/library/code/. The MATLAB code of several other measures are available on MathWorks: https://fr.mathworks.com/matlabcentral/fileexchange/63326-objective-supervised-edge-detection-evaluation-by-varying-thresholds-of-the-thin-edges. These measures can be split into 3 main categories:

- Oversegmentation measures (recording only distances of FPs): Y, Θ, $D^{k}$ and Γ.
- Undersegmentation measure (recording only distances of FNs): Ω.
- Measures recording distances of both FPs and FNs: *H*, $f_{2}d_{6}$, $RDE_{k}$, $\Delta^{k}$, Ψ and λ.

Most of these measures are derived from the Hausdorff distance which is intended to estimate the dissimilarity between each element of two binary images. The λ measure computes a weight for FNs, and, as pointed out in [20], it behaves as expected for shape dissimilarity evaluation. By moving the shape, the score must converge to 0, where the two contours are collocated.

The first video to be compared with the state of the art is video V2, see Figure 5. The table’s texture and borders create FPs at several moments, especially at the end of the video. These FP pixels cause peaks in the score curves (illustrated in Figure A1), particularly from frame 85 to the last frame, unlike the normalized measures, whose scores are shown in Figure 10. They therefore do not determine precisely that the object moves to its target position. Only the undersegmentation measure Ω and the measure λ behave as desired.

**Table A1:** List of error measures involving distances, generally: k=1 or k=2.

Error Measure Name	Formulation	Parameters
Yasnoff measure [24]	$Y\left(G_{t},D_{c}\right)=\frac{100}{\left|I\right|}\cdot \sqrt{\sum_{p\in D_{c}}d_{G_{t}}^{2}\left(p\right)}$	None
Hausdorff distance [25]	$H\left(G_{t},D_{c}\right)=\max\left(\max_{p\in D_{c}}\left(d_{G_{t}}\left(p\right)\right),\max_{p\in G_{t}}\left(d_{D_{c}}\left(p\right)\right)\right)$	None
Maximum distance [3]	$f_{2}d_{6}\left(G_{t},D_{c}\right)=\max\left(\frac{1}{\left|D_{c}\right|}\cdot \sum_{p\in D_{c}}d_{G_{t}}\left(p\right),\frac{1}{\left|G_{t}\right|}\cdot \sum_{p\in G_{t}}d_{D_{c}}\left(p\right)\right)$	None
Distance to $G_{t}$ [3,5,26]	$D^{k}\left(G_{t},D_{c}\right)=\frac{1}{\left|D_{c}\right|}\cdot \sqrt[{k}]{\sum_{p\in D_{c}}d_{G_{t}}^{k}\left(p\right)}$, k=1 for [3,26]	$k\in R^{+}$
Oversegmentation measure [27]	$\Theta \left(G_{t},D_{c}\right)=\frac{1}{FP}\cdot \sum_{p\in D_{c}}{\left(\frac{d_{G_{t}}\left(p\right)}{\delta_{TH}}\right)}^{k}$	for [27]: $k\in R^{+}$ and $\delta_{TH}\in R_{*}^{+}$
Undersegmentation measure [27]	$\Omega \left(G_{t},D_{c}\right)=\frac{1}{FN}\cdot \sum_{p\in G_{t}}{\left(\frac{d_{D_{c}}\left(p\right)}{\delta_{TH}}\right)}^{k}$	for [27]: $k\in R^{+}$ and $\delta_{TH}\in R_{*}^{+}$
RelativeDistanceError [3,7,21,22]	$RDE_{k}\left(G_{t},D_{c}\right)=\sqrt[{k}]{\frac{1}{\left|D_{c}\right|}\cdot \sum_{p\in D_{c}}d_{G_{t}}^{k}\left(p\right)}+\sqrt[{k}]{\frac{1}{\left|G_{t}\right|}\cdot \sum_{p\in G_{t}}d_{D_{c}}^{k}\left(p\right)}$,	$k\in R^{+}$, k=1 for [3], k=2 for [21,22]
Symmetric distance [3,5]	$S^{k}\left(G_{t},D_{c}\right)=\sqrt[{k}]{\frac{\sum_{p\in D_{c}}d_{G_{t}}^{k}\left(p)\right)+\sum_{p\in G_{t}}d_{D_{c}}^{k}\left(p\right)}{\left|D_{c}\cup G_{t}\right|}}$, k=1 for [3]	$k\in R^{+}$
Baddeley’s Delta Metric [28]	$\Delta^{k}\left(G_{t},D_{c}\right)=\sqrt[{k}]{\frac{1}{\left|I\right|}\cdot \sum_{p\in I}{\left|w\left(d_{G_{t}}\left(p\right)\right)-w\left(d_{D_{c}}\left(p\right)\right)\right|}^{k}}$	$k\in R^{+}$ and a convex function w:R↦R
Magnier et al. measure [29]	$\Gamma \left(G_{t},D_{c}\right)=\frac{FP+FN}{\;|G_{t}{|}^{2}}\cdot \;\;\sqrt{\sum_{p\in D_{c}}d_{G_{t}}^{2}\left(p\right)}$	None
Complete distance measure [6]	$\Psi \left(G_{t},D_{c}\right)=\frac{FP+FN}{|G_{t}{|}^{2}}\cdot \;\;\sqrt{\sum_{p\in G_{t}}d_{D_{c}}^{2}\left(p\right)+\;\;\;\sum_{p\in D_{c}}d_{G_{t}}^{2}\left(p\right)}$	None
λ measure [30]	$\lambda \left(G_{t},D_{c}\right)=\frac{FP+FN}{|G_{t}{|}^{2}}\cdot \;\;\sqrt{\sum_{p\in D_{c}}d_{G_{t}}^{2}\left(p\right)+\min\left(|G_{t}{|}^{2},\;\;\frac{|G_{t}{|}^{2}}{TP^{2}}\right)\cdot \sum_{p\in G_{t}}d_{D_{c}}^{2}\left(p\right)}$	None

The scores of state-of-the-art non-normalized measures are also compared to normalized measures using video V3. The main problem, apart from the geometric changes to the object, concern the momentary lack of detection of the edge of the table (horizontal contour crossing the end of video V3, see Figure 6). This contour is not extracted because it appears fuzzy in certain frames and the thresholds used are not necessarily optimized. This disappearance of contours creates FNs compared with the ground truth. Consequently, over-detection measures such as Y, $D^{k}$ and Θ are not disturbed by these FNs, see scores in Figure A2. However, the measures that combine over- and under-detection or only under-detect are seriously disturbed by the occurrence of these FNs (i.e., the disappearance of the horizontal contour). This results in major error peaks in the score curves after 200 frames. For these measures, the scores therefore converge somewhat randomly towards 0, rather than smoothly as they should. These peaks do not occur in the curves for normalized measures. These two examples (V2 and V3) illustrate the importance of normalization, without which FNs or FPs can lead to serious errors.

Regarding video 4 (V4), containing considerable noise that disturbs the edge detection, the tied curves of the different measures are displayed in Figure A3. The Hausdorff measure (*H*) and $\Delta^{k}$ behave stochastically along the video without convergence. Also, on the one hand, Y behaves like $D^{k}$, Θ, $S_{k=2}^{k}$, $RDE_{k=2}$ and $f_{2}d_{6}$, globally decreasing until the half of the video, then stay relatively constant otherwise. On the other hand, Ω, Γ and Ψ stagnate and do not enable analysis of the movement of the shape by visualizing these curves. Lastly, the λ measure behaves as expected with a minimum at the end.
